# Interfacial stabilization for epitaxial CuCrO_2_ delafossites

**DOI:** 10.1038/s41598-020-68275-w

**Published:** 2020-07-09

**Authors:** Jong Mok Ok, Sangmoon Yoon, Andrew R. Lupini, Panchapakesan Ganesh, Matthew F. Chisholm, Ho Nyung Lee

**Affiliations:** 1 Materials Science and Technology Division, Oak Ridge National Laboratory, Oak Ridge, TN 37831 USA; 2 Center for Nanophase Materials Sciences, Oak Ridge National Laboratory, Oak Ridge, TN 37831 USA

**Keywords:** Surfaces, interfaces and thin films, Surfaces, interfaces and thin films

## Abstract

*AB*O_2_ delafossites are fascinating materials that exhibit a wide range of physical properties, including giant Rashba spin splitting and anomalous Hall effects, because of their characteristic layered structures composed of noble metal *A* and strongly correlated *B*O_2_ sublayers. However, thin film synthesis is known to be extremely challenging owing to their low symmetry rhombohedral structures, which limit the selection of substrates for thin film epitaxy. Hexagonal lattices, such as those provided by Al_2_O_3_(0001) and (111) oriented cubic perovskites, are promising candidates for epitaxy of delafossites. However, the formation of twin domains and impurity phases is hard to suppress, and the nucleation and growth mechanisms thereon have not been studied for the growth of epitaxial delafossites. In this study, we report the epitaxial stabilization of a new interfacial phase formed during pulsed-laser epitaxy of (0001)-oriented CuCrO_2_ epitaxial thin films on Al_2_O_3_ substrates. Through a combined study using scanning transmission electron microscopy/electron-energy loss spectroscopy and density functional theory calculations, we report that the nucleation of a thermodynamically stable, atomically thick CuCr_1−x_Al_x_O_2_ interfacial layer is the critical element for the epitaxy of CuCrO_2_ delafossites on Al_2_O_3_ substrates. This finding provides key insights into the thermodynamic mechanism for the nucleation of intermixing-induced buffer layers that can be used for the growth of other noble-metal-based delafossites, which are known to be challenging due to the difficulty in initial nucleation.

## Introduction

*AB*O_2_ delafossite oxides have attracted considerable interest because of their fascinating properties that depend on the choice of *A* and *B* site elements^1–3^. Metallic delafossites, especially PdCoO_2_ and PdCrO_2_, exhibit very high conductivity on the order of 10^–8^ Ω cm with an extremely long mean free path of *l*_m_ ~ 20 μm at low temperatures^4,5^. While the high conductivity and the large spin–orbit coupling associated with huge Rashba splitting^6^ make such metallic delafossites promising candidates for future spintronic devices, their synthesis in thin film forms has not been established. There have been several attempts to grow metallic delafossite thin films, and Pd-based delafossites were recently grown by pulsed laser epitaxy (PLE)^7–9^ and molecular beam epitaxy (MBE)^10,11^. The quality and performance of thin films, however, are not as good as those of single crystals, thus further improvements are needed. The major problem that needs to be overcome is the poor structural quality due to the formation of twin domains and impurity phases^7–11^, which mainly originate from the initial nucleation and structural dissimilarity between the film and substrate. To achieve high quality thin films, therefore, not only is an isostructural substrate or buffer layer with similar lattice parameters needed, but also a deeper understanding on the nucleation and growth mechanisms.

Among various delafossite compounds, Cu-based delafossites are good candidates for resolving the major problems for two reasons. First, the Cu-based delafossites could be utilized as isostructural substrates or buffer layers because of their high resistivity and small lattice mismatch with Pd-based delafossites^1–3^. Second, Cu-based delafossites are known to be readily grown as thin films^12–18^. For example, CuCrO_2_ thin films grown on Al_2_O_3_ substrates are known to be one of the best delafossite thin films with high crystallinity and no impurity phase formation^12–18^. Thus, understanding their growth and nucleation mechanisms can provide guidance on how to grow other delafossite films. Furthermore, we have recently found that deposition of a CuCrO_2_ monolayer before the growth of the PdCrO_2_ thin film significantly reduced the appearance of impurity phases^9^, whereas the formation of a large amount of Cr_2_O_3_ impurity was inevitable for the direct growth of PdCrO_2_ without the buffer layer^9^. In the context of lattice mismatch (δ (%) = (*a*_*s *_– *a*_*f*_)/*a*_*s*_ × 100%, where *a*_*s*_ and *a*_*f*_ are the lattice parameters of the substrate and film, respectively), the role of CuCrO_2_ buffer layer is quite puzzling, because the lattice mismatch of δ = 7.2% between CuCrO_2_ and Al_2_O_3_ is larger than that (δ = 5.9%) of PdCrO_2_ on Al_2_O_3_. Thus, the growth of high-quality CuCrO_2_ thin films cannot be simply understood from conventional thin film growth mechanisms; therefore, a direct observation of the atomic and electronic structure of the CuCrO_2_/Al_2_O_3_ interface is required to reveal the underlying reason for how the epitaxy of CuCrO_2_ on Al_2_O_3_ is accomplished despite the relatively large lattice mismatch.

In this study, we grew high-quality CuCrO_2_ thin films by systematically tuning the growth conditions, including the growth temperature (*T*) and oxygen partial pressure (*P*_O2_). Our films revealed a high crystallinity, smooth surface, and reasonably high resistivity. Using these high-quality CuCrO_2_ thin films, we studied the interface microstructure to understand the nucleation and growth behavior of CuCrO_2_ using scanning transmission electron microscopy (STEM)/electron energy loss spectroscopy (EELS) and density functional theory (DFT) calculations. We have found that atomic-level interfacial intermixing between Al and Cr atoms within the atomic-layer thick substrate surface plays a critical role in stabilizing the nucleation of the CuCrO_2_ delafossite phase. The initial intermixing-induced nucleation seems important to both reduce the most stable impurity phase, Cr_2_O_3_, and to stabilize the high-quality CuCrO_2_ phase.

**Figure 1:**
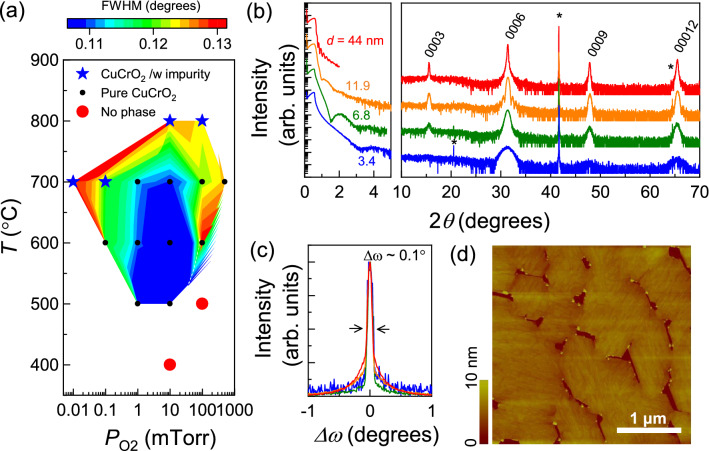
(**a**) Contour plot of full-width at half-maximum (FWHM) of the CuCrO_2_ 0006 peak as a function of oxygen pressure and growth temperature. The blue area corresponds to the optimum growth window, in which CuCrO_2_ thin films exhibit low FWHM values (< 0.1°). (**b**) X-ray reflectivity and X-ray diffraction 2*θ*–*θ* patterns of CuCrO_2_ thin films with different film thicknesses grown under the optimum growth condition, and (**c**) their FWHM of the CuCrO_2_ 0006 peak, exhibiting FWHM ~ 0.1°. (**d**) Atomic force microscopy (AFM) image of a CuCrO_2_ thin film (10 nm in thickness).

To grow CuCrO_2_ thin films with high crystallinity, we mapped out the optimal growth condition for CuCrO_2_ thin films on Al_2_O_3_ (0001) substrates by varying the temperature and oxygen partial pressure using a single-phase CuCrO_2_ target (see Figure S1 in Supplementary Information). Figures S1a,b show X-ray diffraction (XRD) 2*θ*–*θ* scans for CuCrO_2_ films grown under different *T* and *P*_O2_ conditions. The CuCrO_2_ phase could be stabilized under a wide range of growth conditions, but an impurity phase was observed under both low *P*_O2_ (< 0.01 mTorr) and high *T* (> 800 °C) growth conditions. Figure 1a summarizes results for CuCrO_2_ films grown at different *T* and *P*_O2_. The contour plot indicates rocking curve full width at half maximum (FWHM) values of the 0006 CuCrO_2_ peak, and the symbols indicate whether the film is single-phase (black circles) or has impurity phases (blue stars). This result indicates that the growth window for the epitaxy of CuCrO_2_ films is relatively wide (500 < *T* < 800 °C and 0.01 < *P*_O2_ < 500 mT). We found the best quality films were grown at *T* = 650 °C and *P*_O2_ = 10 mTorr.

**Figure 2:**
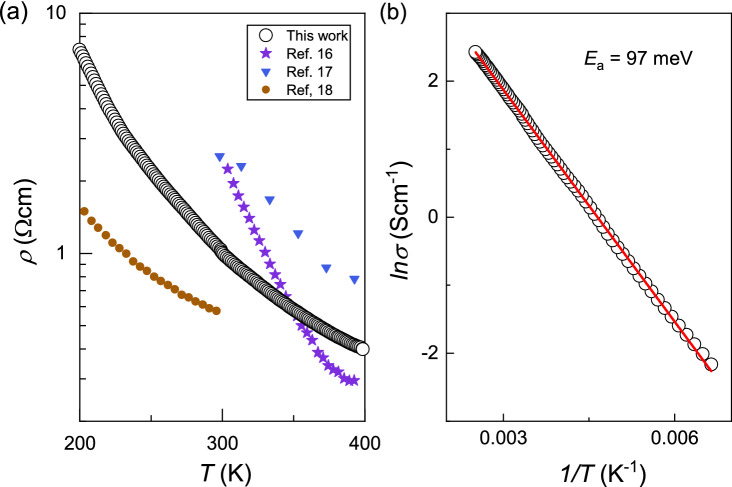
(**a**) Temperature dependence of *dc* resistivity of a CuCrO_2_ thin film, which is compared with films grown by various methods, including PLE^16^, magnetron sputtering^17^, and atomic layer deposition^18^. (**b**) ln *σ* vs. 1/*T* plots for CuCrO_2_ thin film, showing the activation energy of 97 meV.

Figure 1b shows X-ray reflectivity (XRR) and XRD patterns of CuCrO_2_ thin films with different thicknesses (*d* = 3.1–44 nm) grown on Al_2_O_3_ (0001) at the optimum condition (*T* = 650 °C and *P*_O2_ = 10 mTorr). XRR results of all films show clear interference fringes, indicating smooth surfaces of CuCrO_2_ thin films. All of the film peaks in the XRD patterns correspond to the delafossite 0003n peaks. The width of the CuCrO_2_ 0003n peaks become broader with decreasing film thickness, as expected from the Laue function. As shown in Fig. 1c, the rocking curve FWHM values for the 0006 peak of CuCrO_2_ films are ~ 0.1°, which is smaller than those in previous reports. Figure 1d shows an atomic-force microscopy (AFM) image of a CuCrO_2_ thin film surface, showing a triangular shaped grain boundary (we note that such a grain boundary can act as a scattering center in the carrier relaxation process, yielding a higher resistivity than materials without such disorder). The root mean square (RSM) roughness of our film was estimated to be 1.58 nm over a 3 × 3 μm^2^ range. The RSM roughness of a structural domain was only 0.25 nm, which is much smaller than that from spin-coated^12,13^ and MBE-grown films^14^ (RSM = ~ 3–50 nm). The RSM value is even smaller than that from previously PLE grown CuCrO_2_ thin films (RSM = ~ 1 nm)^15^. Figure 2a shows the temperature dependence of 4-probe dc resistivity for a CuCrO_2_ thin film (11.5 nm), which exhibits semiconducting behavior (d*ρ*/d*T* < 0). The thermal activation energy of charge carriers was ~ 97 meV as shown in Fig. 2b, which is consistent with previous reports from thin film samples^16–18^. Overall, the epitaxial growth of high-quality CuCrO_2_ thin films is particularly notable if we consider that the growth of delafossite thin films without impurity phases is a big challenge in many other compounds, e.g. PdCrO_2_, PdCoO_2_, and PtCoO_2_.

**Figure 3:**
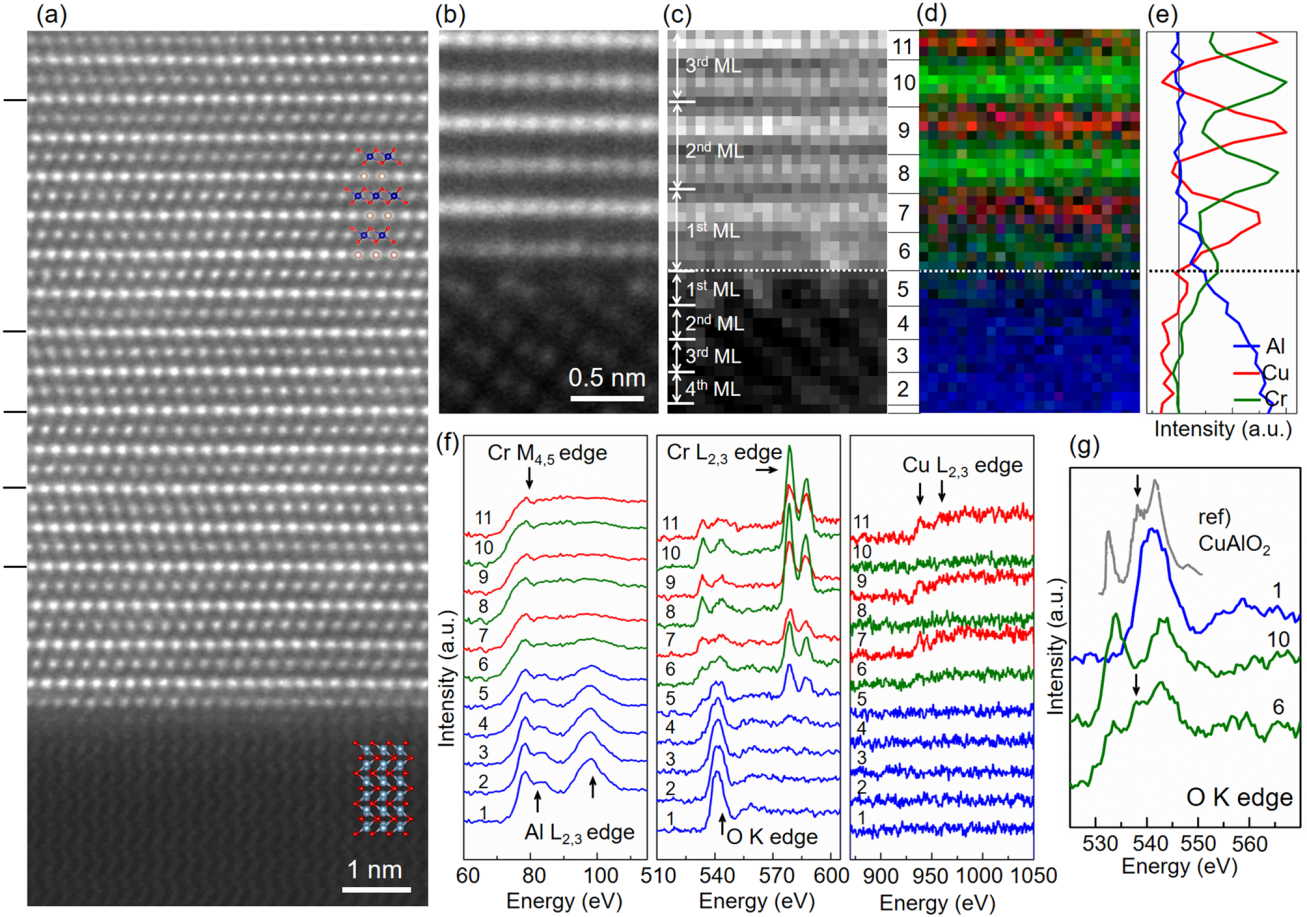
(**a**, **b**) High-angle annular dark field (HAADF) scanning transmission electron microscopy (STEM) image of a CuCrO_2_ thin film grown on an Al_2_O_3_ (0001) substrate seen along the (**a**) [${\overline{1}}100$] and (**b**) [1000] zone axis. (**c**)–(**f**) Electron energy loss spectroscopy (EELS) spectrum imaging of the CuCrO_2_/Al_2_O_3_ interface seen along the [1000] zone axis. The monolayers (MLs) for CuCrO_2_ thin film and Al_2_O_3_ substrate in the (0001) direction were defined as a set of Cu and CrO_2_ sublayers and a single Al_2_O_3_ layer, respectively. (**c**) Simultaneously acquired HAADF–STEM image. (**d**) Color-coded composite elemental map with Al in blue, Cr in green, and Cu in red. (**e**) Integrated line profile of Al, Cr, and Cu signals in (**d**) across the interface. The dotted lines in (**c**)–(**e**) indicate the position of the CuCrO_2_/Al_2_O_3_ interface. (**f**) Layer-resolved integrated EELS spectra of Al–*L*_2,3_, Cr–*M*_4,5_, O–*K*, Cr–*L*_2,3_, and Cu–*L*_2,3_ edges. The position of the atomic layer corresponding to each EELS spectrum is indicated by the numerical index between (**c**) and (**d**). (**g**) EELS O–*K* edge spectra of the Al_2_O_3_ substrate, CuCrO_2_ thin film, and Cr_1−x_Al_x_O_2_ interface layer with an X-ray absorption spectroscopy (XAS) O–*K* edge reference spectrum of CuAlO_2_^30^. It is worth noting that no discernible vacancy-related features could be detected from the integrated line-profile spectra.

**Figure 4:**
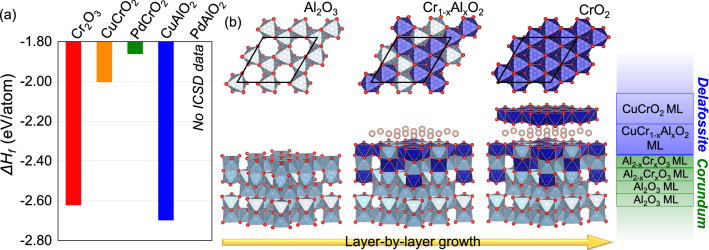
(**a**) Formation enthalpy (Δ*H*_f_) of Cr-based delafossite-related materials estimated by DFT-based fitted elemental-phase reference energies (FERE) method. Structural information of PdAlO_2_ with delafossite symmetry (R${\overline{3}}$m or P6_3_/mmc space group) was considered to compare with other delafossites, but the information is absent in the Inorganic Crystal Structure Database (ICSD). (**b**) Schematics of top and cross-sectional views of a CuCrO_2_/Al_2_O_3_ heterostructure, describing the layer-by-layer growth of a CuCrO_2_ thin film on an Al_2_O_3_ substrate with an atomic-layer thick CuCr_1−x_Al_x_O_2_ nucleation layer. The top view of the top-most surface layer is illustrated on each schematic; Cu atoms are not included in the top view of the surface layer for clear visualization of symmetry matching between delafossite and corundum structures. The MLs of CuCrO_2_ and Al_2_O_3_ are denoted by blue- and green-outlined rectangles on the right side of (**b**), respectively.

Figure 3a shows a high-angle annular dark field (HAADF) STEM image of a CuCrO_2_ thin film grown on an Al_2_O_3_ (0001) substrate seen along the [${\overline{1}}100$] zone axis. The brightest and second brightest features in this image indicate Cu and Cr atomic columns, respectively. Note the HAADF STEM provides scattering intensities that are approximately proportional to the square of the atomic number. Thus, the lightest element, O, is not visible in this HAADF STEM image. The HAADF image confirms that the CuCrO_2_ thin film is epitaxially grown on the Al_2_O_3_ (0001) substrate. The atomic structure at the interface, shown in Fig. 3a,b, exhibits several interesting aspects. First, the growth of the CuCrO_2_ thin film initiates with the CrO_2_ sublayer, followed by the Cu sublayer. Second, the top one or two layers of the Al_2_O_3_ substrate exhibit a brighter intensity than the bulk layers of the substrate. In this study, a monolayer (ML) of each material was defined to satisfy stoichiometry. That is, the MLs for CuCrO_2_ thin film were composed of a set of Cu and CrO_2_ sublayers (thickness: 0.57 nm) and for Al_2_O_3_ substrate in the (0001) direction as a single Al_2_O_3_ layer (thickness: 0.22 nm), which are illustrated in Fig. 4b. Thus the increased intensity implies that some atomic-level interfacial intermixing occurred during the initial stage of film growth. Although it was not identified previously, these interfacial features were also similarly observed in a PdCrO_2_ thin film grown on a CuCrO_2_-buffered Al_2_O_3_ (0001) substrate^9^. In addition, the HAADF STEM image shows that stacking faults exist in the delafossite CuCrO_2_ thin film, denoted by black lines on the left side of Fig. 3a. Such stacking faults were frequently observed in previous PLE or MBE grown delafossite films^9,10^. The presence of these stacking faults indicates that their formation energies are relatively small in this delafossite film. Further studies will be required to understand the influence of stacking faults on the optical and transport properties of delafossite materials.

To systematically understand the interface structure and the nucleation of epitaxial growth of CuCrO_2_ thin films, EELS spectrum imaging was performed across the CuCrO_2_/Al_2_O_3_ interface as shown in Fig. 3c–g. Previous studies have shown that the direct interpretation of interface structure can be achieved only under very thin specimen conditions (ideally less than approximately 20 nm)^19,20^. Otherwise, complex propagation effects, which include beam broadening, cross-talk, and dechanneling, result in the potential misinterpretation of interface structure^21,22^. For this study, we selected a thin region of a specimen for atomic-scale quantification of the interface structure. Thickness measurements across the interface using low-loss EELS spectra are shown in Figure S2 of the Supplementary Information. We confirmed by the 2D spectrum line profile that core-loss excitations are sensitive to the individual atomic planes (see Figure S3 in Supplementary Information). The O–*K* and Cr–*L*_*2,3*_ edge signals dropped to almost zero at the Cu sublayers, as did the Cu–*L*_*2,3*_ edge at the CrO_2_ sublayers. Figure 3c shows a HAADF STEM image of the CuCrO_2_/Al_2_O_3_ interface, which is simultaneously acquired during EELS spectrum imaging. Figure 3d,e show a color-encoded elemental map and an elemental line profile, respectively, across the interface with Al in blue, Cr in green, and Cu in red. Figure 3f shows layer-resolved integrated EELS spectra of Al–*L*_*2,3*_, Cr–*M*_*4,5*_, O–*K*, Cr–*L*_*2,3*_, and Cu–*L*_*2,3*_ edges. Figure 3g highlights a collection of O–K edge spectra of the Al_2_O_3_ substrate, CuCrO_2_ thin film, and CuCrO_2_/Al_2_O_3_ interface. The position of the atomic layer corresponding to each spectrum in Fig. 3f,g is denoted in between Fig. 3c,d. For elemental mapping and the line profile, the second peak of Al–*L*_*2,3*_ edge, Cr–*L*_*2,3*_ edge, and Cu–*L*_*2,3*_ edge signals in Fig. 3f were used; because the first peak of Al–*L*_*2,3*_ edge overlaps with the Cr–*M*_*4,5*_ edge signal. To minimize specimen damage due to electron beam irradiation, EELS spectrum imaging was performed using a low-current electron probe with a short exposure time (0.01 s per pixel).

Even in the thinnest specimen section, both Cr and Al elements are clearly detected across the interface as shown in Fig. 3d,e, suggesting interlayer mixing occurred at the interface during the film growth. We note that the plume energy in PLE growth is quite high (1–100 eV)^23^, exceeding the surface bonding energy of substrate materials (typically on the order of 1 eV). Thus, the growth species can easily penetrate into the substrate surface^23,24^, resulting in intermixing of elements especially under high vacuum conditions. Interestingly, the concentration of Cr was significantly reduced only for the first ML of the CuCrO_2_ thin film. The stoichiometry of the CuCrO_2_ layer appears to be fully recovered from the second ML of the film, indicating that the interfacial inter-layer-mixing in the thin film largely occurred within one ML. The Cr–*L*_*2,3*_ edge spectra in Fig. 3f further confirmed that Cr atoms penetrated up to two MLs below the interface, and the oxidation state of Cr ions was maintained as Cr^3+^ even in the Al_2_O_3_ substrate side (the shape and energy of the Cr–*L*_*3*_ edge do not change across the interface^25,26^). Meanwhile, the Cu–*L*_*2,3*_ edge was not detected on the substrate side, verifying that Al atoms were intermixed with the *B*-site Cr atoms not Cu atoms. In general, the O–*K* edge in transition metal oxides has been used for the investigation of the individual electronic structure of materials, since the 1s core states have a relatively small exchange interaction with the final states, resulting in no visible multiplet effects^27–29^. The O–*K* edge spectra profile across the interface confirms that one ML above the interface and two MLs below the interface exhibits the characteristic electronic structures from those of the CuCrO_2_ thin film and Al_2_O_3_ substrate, respectively. More interestingly, the O–*K* edge of the first ML of the CuCrO_2_ thin film (spectrum #6) revealed the same spectral signature as that of the CuAlO_2_ delafossite, which is shown in Fig. 3g. The overall shape of the O–*K* edge of the first ML is similar to that of CuAlO_2_^30^, and, in particular, the peak indicated by the black arrow, which cannot be explained by the O–*K* edge spectra of the existing CuCrO_2_ and Al_2_O_3_, only appears in the O–*K* edge of the first ML. The in-depth analysis of EELS spectral images strongly suggests that the interfacial intermixing, which occurred during the initial stage of the epitaxial growth, creates an atomically thin ML of CuCr_1−x_Al_x_O_2_ alloy.

To evaluate the thermodynamic stability of delafossites compared to the Cr_2_O_3_ impurity phase, the formation enthalpy (Δ*H*_f_) was calculated using the DFT-based fitted elemental-phase reference energies (FERE) method^31^. As shown in Fig. 4a, Δ*H*_f_ of Cr-based delafossites, including CuCrO_2_ and PdCrO_2_, were far greater than that of Cr_2_O_3_, accounting for the formation of Cr_2_O_3_ impurities. Interestingly, CuAlO_2_ was found to be much more stable than CuCrO_2_ and even more stable than Cr_2_O_3_; 0.08 eV/atom lower than that for Cr_2_O_3_. These numerical results suggest that the substitution of Al atoms for Cr atoms in CuCrO_2_ will lower its formation enthalpy. This thermodynamic consideration explains the formation of the CuCr_1−x_Al_x_O_2_ interfacial ML we observed in our STEM–EELS investigations. In addition, the interfacial mixing helps to reduce the epitaxial strain as the lattice constant of CuCr_1−x_Al_x_O_2_ is closer to the Al_2_O_3_ substrate than for pure CuCrO_2_. It is worth noting that the formation of PdAlO_2_ has not been experimentally reported so far, suggesting that the substitution of Al atoms for Cr atoms to form the equivalent interfacial PdCr_1−x_Al_x_O_2_ for the growth of epitaxial PdCrO_2_ on Al_2_O_3_ is unlikely.

The STEM–EELS and DFT results provide direct insights into the nucleation of epitaxial growth of CuCrO_2_ thin films and the critical role of a CuCrO_2_ buffer layer for the epitaxy of delafossites. First, inter-layer-mixing was observed at the interface, but not further into the film, indicating that it happens at the initial nucleation step of the epitaxial growth. At the initial nucleation stage, Cr atoms will penetrate into the sub-surface-layers of the Al_2_O_3_ substrate and, as an exchange, Al atoms will out-diffuse to the surface. Second, the free Al atoms at the surface will act to stabilize the nucleation of CuCrO_2_ delafossite thin films. Without these Al atoms, the Cr_2_O_3_ will be the most stable phase at the nucleation step, disturbing the formation of the delafossite phase. Third, the homogenous and stable nucleation, with the delafossite symmetry provided by CuCr_1−x_Al_x_O_2_, will enable the high-quality growth of CuCrO_2_ thin films. The growth process understood here is summarized in Fig. 4b. In a previous study, we reported that the use of a one-ML-thick CuCrO_2_ buffer layer significantly suppressed the formation of Cr_2_O_3_ impurities in the epitaxy of PdCrO_2_ thin films^9^. It was quite puzzling because the lattice mismatch of the CuCrO_2_/Al_2_O_3_ interface (δ = 7.2%) is much larger than that of the PdCrO_2_/Al_2_O_3_ interface (δ = 5.9%), which is counter intuitive. Our discovery of the formation of the CuCr_1−x_Al_x_O_2_ delafossite at the CuCrO_2_/Al_2_O_3_ interface now explains the role of the CuCrO_2_ buffer layer in the growth of the PdCrO_2_ thin films. The deposition of CuCrO_2_ buffer layer will induce the homogenous and stable nucleation with delafossite symmetry, which cannot be achieved from the direct deposition of PdCrO_2_ layers. Note again that the preferential nucleation by Al substitution only occurs for Cu-based delafossites, not for Pd-based delafossites; the substitution of Al atoms for Cr atoms acts to decompose PdCrO_2_ delafossite films.

In summary, we have grown high quality CuCrO_2_ thin films by pulsed laser epitaxy. Compared with CuCrO_2_ thin films grown by other methods, PLE grown films show better quality in terms of crystallinity and surface roughness. The successful growth was possible owing to the non-equilibrium energetic process of PLE growth. The intermixing-induced alloying of the Al and Cr atoms was found to play a crucial role in stabilizing the nucleation of CuCrO_2_ delafossite phase and in reducing the most stable impurity phase Cr_2_O_3_ often found in other delafossites. We believe a similar consideration can be also applied to Co-based delafossites as the formation of the Co_3_O_4_ spinel structure has been also a challenge for, e.g., PdCoO_2_^7,8,10,11^. Our results suggest that the key to achieving the layer-by-layer growth of CuCrO_2_ delafossite films is the nucleation of the structurally similar CuCr_1−x_Al_x_O_2_ buffer layer at the interface when grown on structurally dissimilar substrates, e.g., conventional Al_2_O_3_ substrates. Thus, this discovery may provide a critical strategy for the epitaxial growth of other delafossites with the new CuCr_1−x_Al_x_O_2_ buffer layer to accelerate innovations in future electronic and spintronic quantum devices made from delafossites.

## Methods

### Thin film growth

High quality CuCrO_2_ thin films were grown on *c*-plane Al_2_O_3_ substrates by PLD using a polycrystalline CuCrO_2_ target. The polycrystalline CuCrO_2_ is prepared by sintering the mixture of Cu_2_O and Cr_2_O_3_ at 1,100 °C for 10 h in air. The obtained pure polycrystalline CuCrO_2_ were pelletized and annealed at 800 °C. Before the thin film growth, commercially available Al_2_O_3_ (0001) substrates (CrysTec, Germany) were annealed at 1,100 °C for 1 h to achieve atomically flat surfaces with step-terrace structure. For the CuCrO_2_ film growth, the growth conditions were widely varied (*T* = 400–800 °C, *P*_O2_ = 0.01–500 mTorr), whereas the repetition rate and energy of the KrF excimer laser (*λ* = 248 nm) were fixed at 5 Hz and 1.5 J/cm^2^, respectively. The best CuCrO_2_ epitaxial thin films were obtained at optimal growth conditions of *T* = 650 °C, *P*_O2_ = 10 mTorr. After the growth, the samples were cooled to room temperature in *P*_O2_ = 100 Torr.

### Characterization

The crystal structure was characterized by X-ray diffraction (XRD) using a four-circle high-resolution X-ray diffractometer (X’Pert Pro, PANalytical; Cu *Kα*_1_ radiation), and the thickness of the film (*d*) was calibrated using X-ray reflectivity (XRR). The surface morphology measurements were made with atomic force microscopy (Veeco Dimension 3100). Cross-sectional TEM specimens were prepared using low-energy ion milling at LN_2_ temperature after mechanical polishing. HAADF STEM measurements were performed on Nion UltraSTEM200 operated at 200 kV. The microscope is equipped with a cold field emission gun and a corrector of third- and fifth-order aberration for sub-Angstrom resolution. The convergence half-angle of 30 mrad was used and the inner angle of the HAADF STEM was approximately 65 mrad. To minimize the electron-irradiation damage during EELS mapping, EELS spectra were measured at 0.01 s collection time.

### Estimation of the enthalpy of formation (*ΔH*_*f*_) using ab-initio DFT calculations

Ab initio DFT calculations were performed using the Vienna ab initio simulation package (VASP) code^32^, and the enthalpy of compound formation is estimated using the fitted elemental-phase reference energies (FERE) method^31^. The Perdew–Burke–Ernzerhof plus Hubbard correction (PBE + U) was used for the exchange–correlation functional^33^, in which the double-counting interactions were corrected using the full localized limit (FLL)^34^. The Hubbard U parameter of 3 eV (U = 3 eV) is used for all transition metals except Cu for U = 5 eV following previous work^31^. A plane wave basis set at a cutoff energy of 600 eV was used to expand the electronic wave functions, and the valence electrons were described using the projector-augmented wave potentials. The Γ-centered 9 × 9 × 9 Monkhorst–Pack K-point grid was used for sampling the Brillouin zone. Input structures are obtained from the Inorganic Crystal Structure Database (ICSD) and all cells and atomic positions in our calculations were relaxed with the force criteria of 0.01 eV/Å. In the FERE approach, the enthalpy of formation (*ΔH*_*f*_) of a chemical compound A_n1_B_n2_ … is expressed by the following equation:

$$\Delta H_{f} \left( {A_{n1} B_{n2} \cdots } \right) = E_{tot}^{DFT} \left( {A_{n1} B_{n2} \cdots } \right) - \mathop \sum \limits_{i} n_{i} \mu_{i}^{DFT} - \mathop \sum \limits_{i} n_{i} \delta \mu_{i}^{FERE}$$

where $E_{tot}^{DFT}$ is the total energy per formula unit of a given compound, $\mu_{i}^{DFT}$ are the total energies per atom of the elements in their elemental reference phase, and $\delta \mu_{i}^{FERE}$ are the FERE correction energies of the elements. The $\delta \mu_{i}^{FERE}$ for 50 chemical elements are tabulated in the paper describing the FERE approach^31^.

## Supplementary information

Supplementary Information.
