# Author Correction: Interfacial stabilization for epitaxial CuCrO_2_ delafossites

**DOI:** 10.1038/s41598-023-31162-1

**Published:** 2023-03-14

**Authors:** Jong Mok Ok, Sangmoon Yoon, Andrew R. Lupini, Panchapakesan Ganesh, Matthew F. Chisholm, Ho Nyung Lee

**Affiliations:** 1 Materials Science and Technology Division, Oak Ridge National Laboratory, Oak Ridge, TN 37831 USA; 2 Center for Nanophase Materials Sciences, Oak Ridge National Laboratory, Oak Ridge, TN 37831 USA

Correction to: *Scientific Reports* https://doi.org/10.1038/s41598-020-68275-w, published online 09 July 2020

The original version of this Article contained an error in Figure 3, panel (a), where the positioning of the atomic structure overlaid on the HAADF STEM image was incorrect.

The original Figure 3 and its accompanying legend appear below.

**Figure 3:**
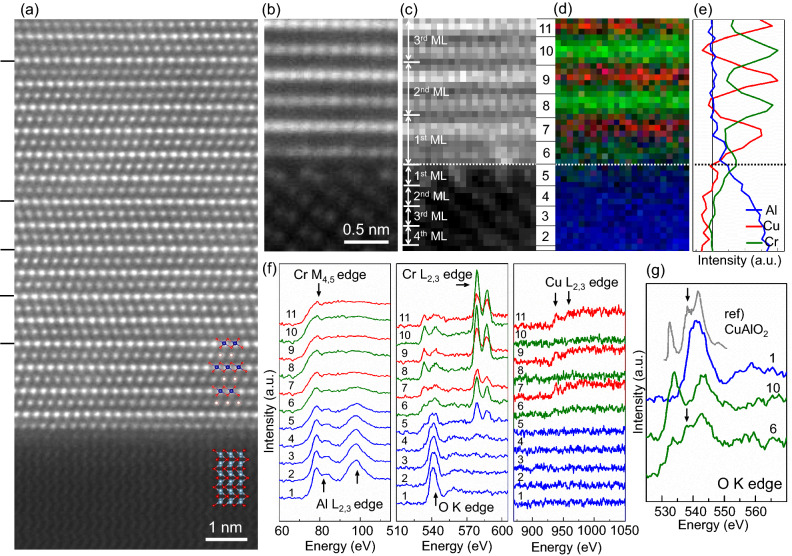
(**a**, **b**) High-angle annular dark field (HAADF) scanning transmission electron microscopy (STEM) image of a CuCrO_2_ thin film grown on an Al_2_O_3_ (0001) substrate seen along the (**a**) [$\overline{1}100$] and (**b**) [1000] zone axis. (**c**)–(**f**) Electron energy loss spectroscopy (EELS) spectrum imaging of the CuCrO_2_/Al_2_O_3_ interface seen along the [1000] zone axis. The monolayers (MLs) for CuCrO_2_ thin film and Al_2_O_3_ substrate in the (0001) direction were defined as a set of Cu and CrO_2_ sublayers and a single Al_2_O_3_ layer, respectively. (**c**) Simultaneously acquired HAADF–STEM image. (**d**) Color-coded composite elemental map with Al in blue, Cr in green, and Cu in red. (**e**) Integrated line profile of Al, Cr, and Cu signals in (**d**) across the interface. The dotted lines in (**c**)–(**e**) indicate the position of the CuCrO_2_/Al_2_O_3_ interface. (**f**) Layer-resolved integrated EELS spectra of Al–*L*_2,3_, Cr–*M*_4,5_, O–*K*, Cr–*L*_2,3_, and Cu–*L*_2,3_ edges. The position of the atomic layer corresponding to each EELS spectrum is indicated by the numerical index between (**c**) and (**d**). (**g**) EELS O–*K* edge spectra of the Al_2_O_3_ substrate, CuCrO_2_ thin film, and Cr_1−x_Al_x_O_2_ interface layer with an X-ray absorption spectroscopy (XAS) O–*K* edge reference spectrum of CuAlO_2_^30^. It is worth noting that no discernible vacancy-related features could be detected from the integrated line-profile spectra.

The original Article has been corrected.

