# Supplementary material for: Interfacial stabilization for epitaxial CuCrO2 delafossites
Source: Sci Rep. 2020 Jul 9;10:11375. doi: 10.1038/s41598-020-68275-w (PMC7347942; doi:10.1038/s41598-020-68275-w)
Supplement: Supplementary file 1 — Supplementary Information. [file 41598_2020_68275_MOESM1_ESM.docx]

Supplementary Information

**Interfacial stabilization for epitaxial CuCrO_2_ delafossites**

Jong Mok Ok^1,†^, Sangmoon Yoon^1,†^, Andrew R. Lupini^2^, Panchapakesan Ganesh^2^, Matthew F. Chisholm^2^, Ho Nyung Lee^1^*

^1^Materials Science and Technology Division, Oak Ridge National Laboratory, Oak Ridge, TN 37831, U.S.A.

^2^Center for Nanophase Materials Sciences, Oak Ridge National Laboratory, Oak Ridge, TN 37831, U.S.A.

**
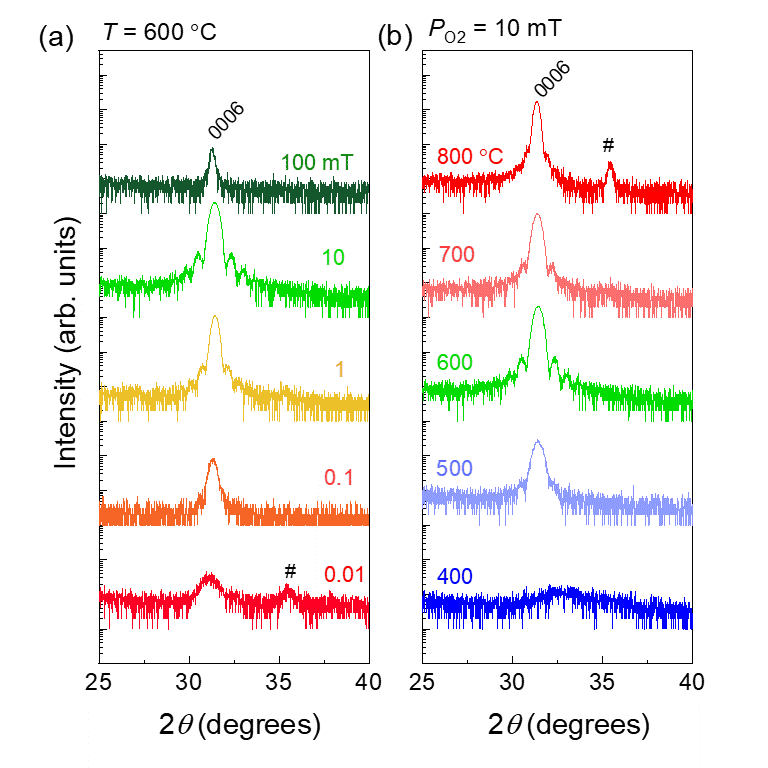
**

**Figure S1. Growth condition dependent X-ray diffraction.** (a-b) XRD 2*θ-θ* scans of CuCrO_2_ thin films grown on Al_2_O_3_ substrate about the 0006 delafossite peak as functions of (a) oxygen pressure at *T* = 700 ̊C and (b) growth temperature at *P*_O2_ = 10 mTorr.


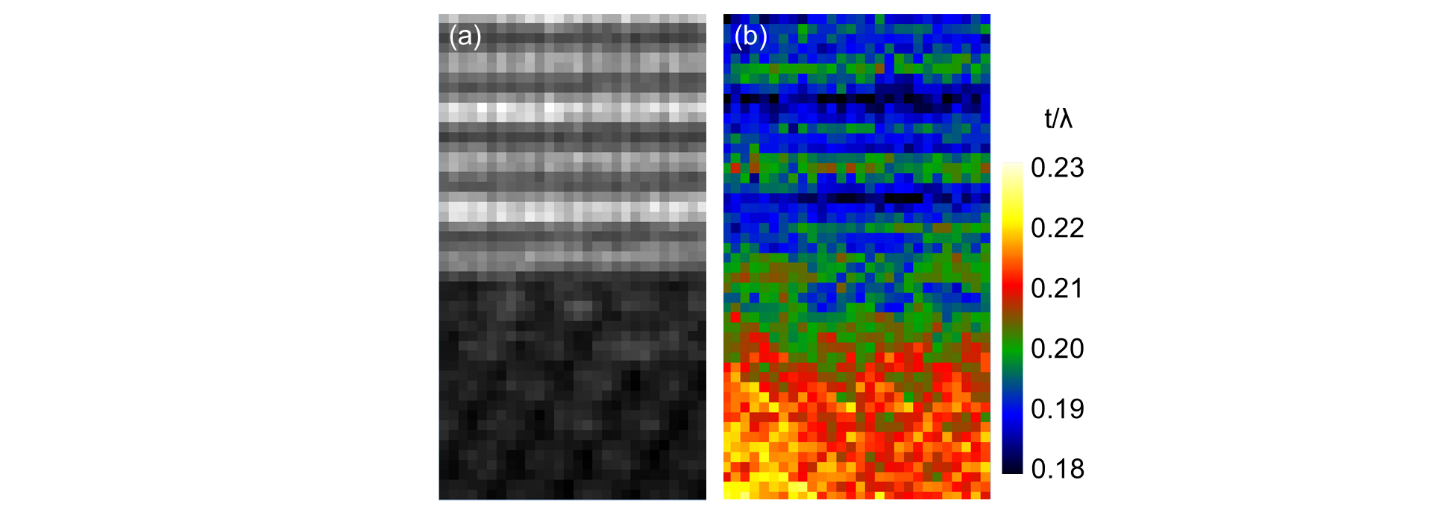


**Figure S2. Thickness measurements across the CuCrO_2_/Al_2_O_3_** **interface using low-loss EELS.** (a)-(b) low-loss EELS spectrum imaging of the CuCrO_2_/Al_2_O_3_ interface seen along the [1000] zone axis. (c) Simultaneous acquired HAADF STEM image. (d) Relative thickness (t/λ) map with temperature color scale where t is the specimen thickness and λ is the mean free path of the sample. Roughly, t/λ of 0.1 indicates a thickness of about 10nm. Thus t/λ ≲ 0.2 indicates that the TEM specimen is still in a thin condition.


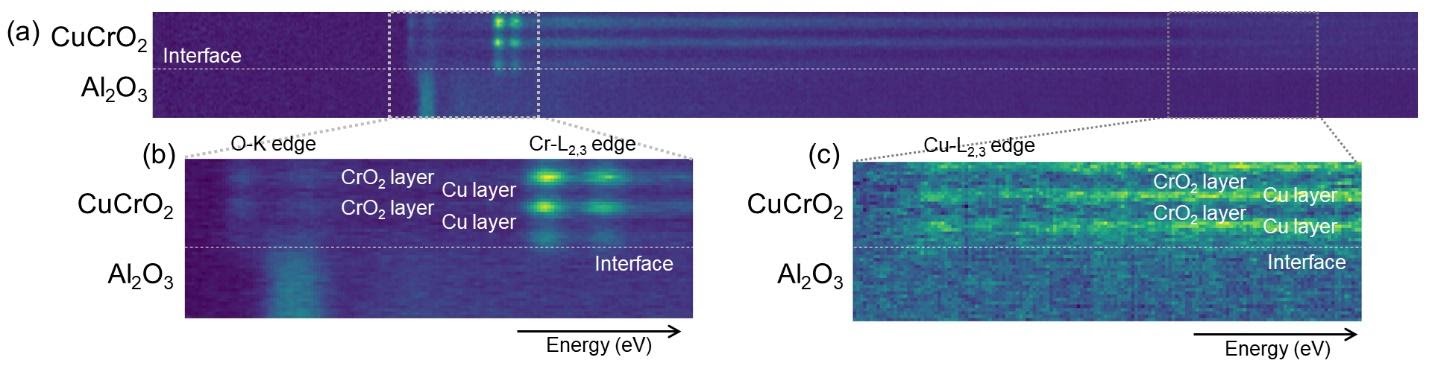


**Figure S3. 2D spectrum line profile across the CuCrO_2_/Al_2_O_3_** **interface.** (a) Full range of 2D spectrum line profile from O–*K* edge to Cu–*L_2,3_* edge. (b)-(c) Magnified view of 2D spectrum line profile for (b) O–*K* edge and Cr–*L_2,3_* edge and (c) Cu–*L_2,3_* edge. Spectrum line profile shows that atomic plane resolution is achieved in CuCrO_2_. As shown in Fig. S3 (b) and (c), the O–K and Cr–*L_2,3_* edge and Cu–*L_2,3_* edge signals drop to almost zero at the Cu and CrO_2_ sublayers of the CuCrO_2_ thin film, respectively.


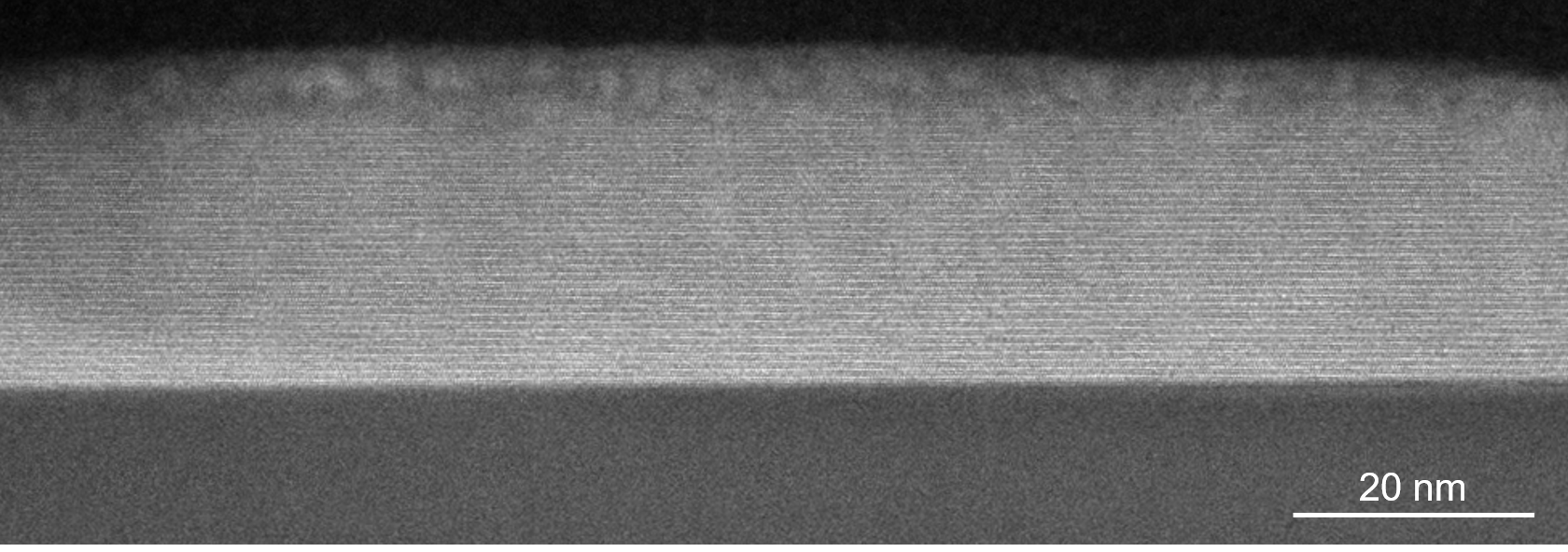


**Figure S4. Low-magnification HAADF STEM image of a CuCrO_2_ thin film grown on an Al_2_O_3_(0001) substrate seen along the [**$\bar{\boldsymbol{1}}$**100] zone axis.** This low-magnification image verifies that the CuCrO_2_ thin film grown on the Al_2_O_3_ substrate has a sharp interface without any other impurity phases. The top surface of the CuCrO_2_ thin film is observed to be amorphized from damage during the ion milling process.
